# Sonographic Estimation of the Fetal Head Circumference: Accuracy and Factors Affecting the Error

**DOI:** 10.1007/s13224-021-01574-y

**Published:** 2021-10-09

**Authors:** Vidyashree Ganesh Poojari, Aiswarya Jose, Muralidhar V. Pai

**Affiliations:** Department of Obstetrics and Gynecology, Kasturba Medical College, Manipal, Manipal Academy of Higher Education (MAHE), Manipal, Karnataka India

**Keywords:** Fetal head circumference, Postnatal head circumference, Accuracy, Error

## Abstract

**Background:**

Sonographic measurement of fetal head circumference (HC) is an essential parameter for the estimation of fetal weight as well as in cases with abnormal fetal head size. Since there is a lack of data, the present study was to assess the accuracy of ultrasonographic estimation of fetal HC and to identify factors that affect the accuracy of fetal HC estimation.

**Material and Methods:**

A prospective cohort observational study was conducted for a year. Sonographic fetal biometry including HC was performed, and fetal HC was measured postnatally. Measures of accuracy and various factors which affect the accuracy are analyzed.

**Results:**

Ultrasonographic HC underestimated actual postnatal HC in 87.5% and overestimated actual HC in 12.5%. Sonographic underestimation of HC persisted throughout gestation and became more pronounced as gestational age increased. Error in HC was statistically significant in those with low liquor and anterior placenta and in those who had instrumental delivery. Parity, fetal presentation, and maternal diabetes did not affect the error in ultrasonographic measurement of head circumference. When the HC was beyond 95th centile on ultrasound, the error detected postnatally was significant (− 14 mm vs. − 8 mm), though not statistically significant (*p* value 0.82). The difference between the sonographic and postnatal HC was also related to the mode of delivery with the highest error seen in those who had instrumental vaginal delivery (*p* value 0.031).

**Conclusion:**

The ultrasound estimation of fetal HC is associated with significant underestimation of the actual HC measured postnatally. The error in measuring fetal HC increased in those with advanced gestational age, low liquor, and anterior location of the placenta and in those who had instrumental vaginal delivery. The measurement error may have important implications in specific clinical scenarios like monitoring pregnancy with fetal growth restriction, suspected fetal head growth abnormalities, and labor outcome.

## Introduction

Assessment of fetal biometry by ultrasound is an essential and universal part of antenatal care, not only in managing high-risk pregnancies and growth monitoring but also vital in labor outcome [1]. Sonographic measurement of fetal head circumference (HC) is an essential parameter for the estimation of fetal weight as well as in cases with abnormal fetal head size (i.e., microcephaly/macrocephaly) [2, 3]. Lately, studies have shown that fetal head circumference is more important than fetal weight in the labor outcome [4–6]. Hence, measuring fetal head circumference is of utmost importance with precision. However, few studies are found regarding the accuracy of sonographic estimation of HC compared with actual postnatal HC during the literature search. Some studies found that the sonographic head circumference underestimated actual HC, while others found that the difference was statistically insignificant [7]. There is also a lack of data on factors affecting the accuracy of estimation of ultrasonographic fetal HC.

Our study aimed to assess the accuracy of ultrasonographic estimation of fetal HC in a cohort of women undergoing sonographic examination within 5 days before delivery and also to identify factors that affect the accuracy of ultrasonographic estimation of fetal HC.

## Materials and Methods

The present study was a prospective cohort observational study conducted for a year in a tertiary care referral hospital. After informed written consent, we considered antenatal history, demographic characteristics, gestational age at delivery, and mode of delivery. Certain factors which may affect the accuracy like parity, fetal presentation, placental location, amniotic fluid index (AFI), ultrasonographic HC > 95th centile, and diabetic status were analyzed.

Inclusion criteria for the study were all antenatal women with a singleton pregnancy, those who delivered live babies with gestational age beyond 28 weeks. We excluded multiple pregnancies, cases in which there was uncertainty regarding gestational age, and anomalous babies from the study.

**Fig. 1:**
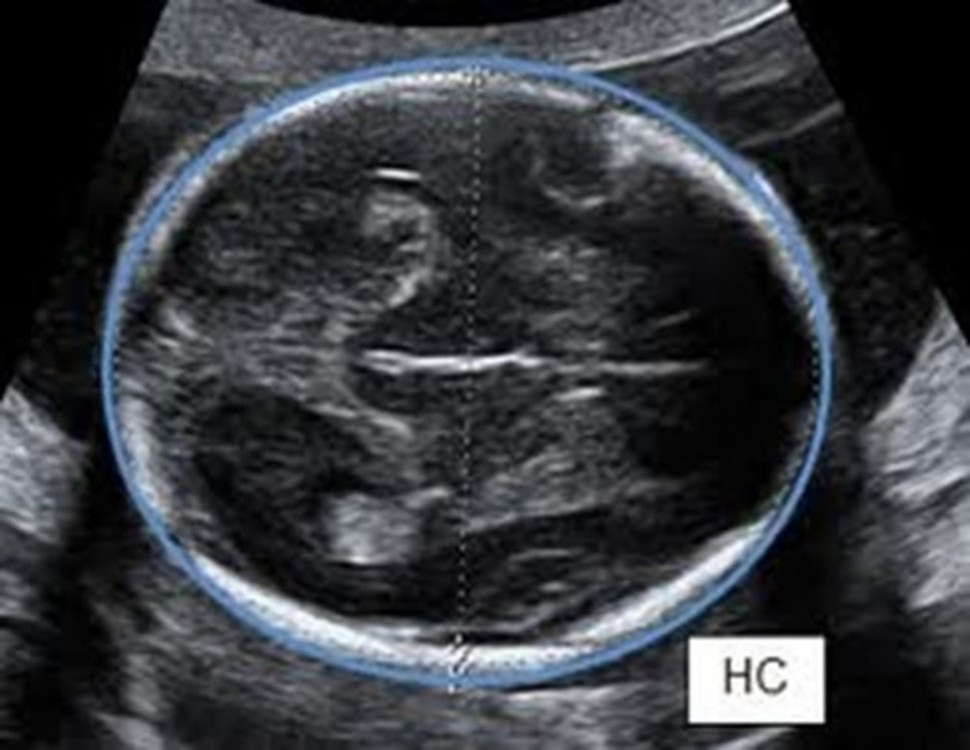
Ultrasonographic image of measuring fetal head circumference

A senior consultant in the ultrasound unit of the obstetrics and gynecology department performed a detailed growth scan within 5 days of delivery—fetal biometry measured as per the ISUOG guidelines [8]. The HC was measured as an ellipse around the perimeter of the fetal skull on an axial plane that traverses the thalami and cavum septum pellucidum. The transducer must be perpendicular to the central axis of the head, so that the hemispheres and calvaria should appear symmetric. Cerebellar hemispheres should not be in the plane of the image. Ellipse was traced at the outer skull border (Fig. 1).

**Fig. 2:**
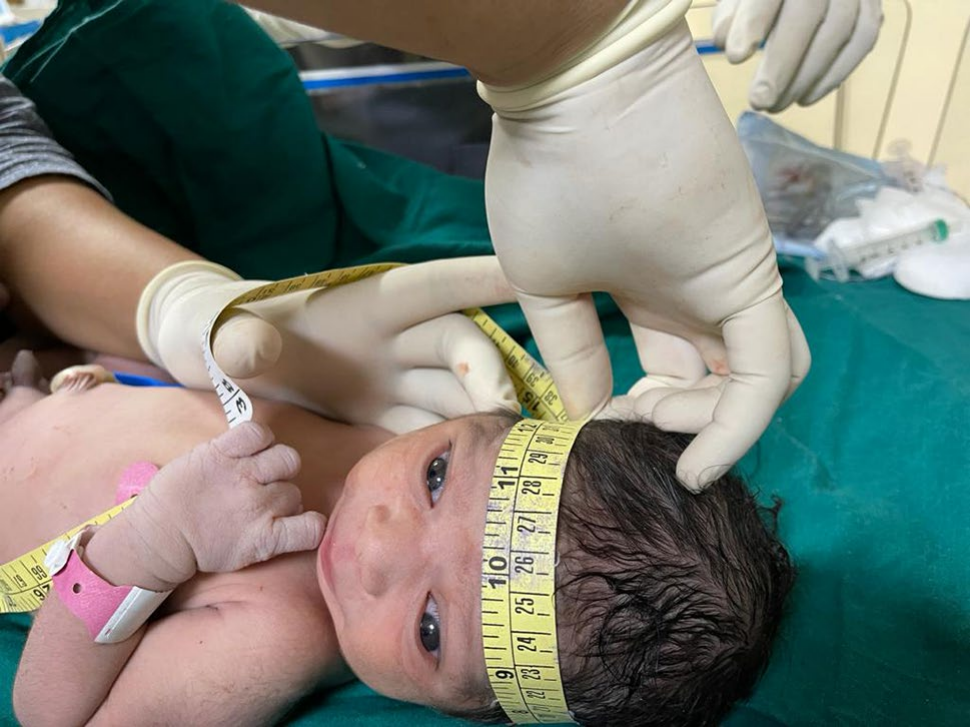
Measuring head circumference of the new born using a measuring tape

The actual postnatal head circumference was measured by a trained pediatrician 3 days after delivery using a measuring tape over the supraorbital ridge and occipital prominence (Fig. 2). This delay of 3 days minimized the measurement error caused by scalp edema, caput succedaneum. Sonographic estimations of fetal HC were compared with the actual HC measured postnatally, and then the accuracy measures were calculated. Mean simple error is the difference between the ultrasonographic HC and postnatal HC. The study was approved by the institutional ethical review board (IEC 514/2017).

## Statistical Analysis

We performed data analysis using SPSS 20 software. One sample *t* test is used to determine the sample size. While comparing measures of accuracy, one-way ANOVA was used for the mean simple error. Univariate logistic regression analysis was used to analyze various factors affecting the errors in measurement of fetal HC. Pearson’s correlation coefficient was used to determine significance in the correlation of various parameters with actual HC. *p *value was significant if differences were < 0.05.

## Results

A total of 242 women met the inclusion criteria for the study during the study period, out of which 216 (89.25%) were recruited for the study and analyzed. The remaining 26 (10.7%) were unwilling to participate in the study or lost to follow-up.

Table 1 shows the demographic characteristics of the study group. The mean age of the antenatal women was 26.85 ± 3.7 years. The mean gestational age in weeks was 36.8 ± 2.8. The mean ultrasonographic HC in millimeters (mm) was 315.3 ± 19.1. The mean actual postnatal HC was 323.7 ± 28.8 mm. The mean estimated fetal weight on ultrasound was 2711 ± 642 g. The mean actual birth weight was 2624 ± 595 g. Fifteen (6.9%) women had diabetes in pregnancy.

**Table 1:** Demographic characteristics of the study population (*N* = 216)

Characteristics	Mean ± SD
Age (years)	26.85 ± 3.7
Gestational age (weeks)	36.8 ± 2.8
Ultrasonographic HC (mm)	315.3 ± 19.1
Actual postnatal HC (mm)	323.7 ± 28.8
Ultrasonographic estimated fetal weight (grams)	2711 ± 642
Actual birth weight (grams)	2624 ± 595
Diabetes in pregnancy	15 (6.9%)

Overall, the mean sonographic fetal HC was lower than the postnatal HC (Table 1). The tendency for sonographic underestimation of HC persisted throughout gestation and became more distinct as gestational age increased, reaching a mean difference of − 8.9 mm at gestational age beyond 37 weeks (Table 2). Ultrasonographic HC underestimated actual postnatal HC in 189 cases (87.5%) and overestimated actual HC in 27 cases (12.5%).

**Table 2:** Factors contributing to the accuracy of measuring fetal head circumference (*N* = 216)

Variables	*N* = 216	Mean simple error (mm)	*p* value*
*Parity*			
Primigravida	148	− 6.5	0.123
Multigravida	68	− 9.4	
*Gestational age (weeks)*			
28–33^+6^	32	− 5	0.035
34–36^+6^	53	− 8.1	
> 37	131	− 8.9	
*Diabetes in pregnancy*			
Present	15	− 13.0	0.407
Absent	201	− 8.03	
*Presentation*			
Vertex	201	− 8.40	0.507
Breech	15	− 8.00	
*Placental location*			
Anterior	70	− 9.3	0.040
Posterior	128	− 8.4	
Fundal	18	− 4.2	
*Amniotic fluid index*			
8–25	160	− 7.4	0.056
5–8	43	− 8.4	
< 5	12	− 12	
*HC* > *95th centile*			
Present	13	− 14	0.825
Absent	203	− 8	
*Mode of delivery*			
Normal	105	− 9.4	0.031
Instrumental	3	− 13.5	
Cesarean	108	− 7.1	

*p value < 0.05 is considered significant

Table 2 presents various demographic, obstetric, and sonographic parameters that were thought to have a potential effect on HC error. The error in HC measurement and the degree of underestimation were significantly higher in gestational age beyond 34 weeks, with an error of 8.1 mm between 34 and 36^+6^ weeks and 8.9 mm beyond 37 weeks. Error in HC was significantly more in those with the anterior location of the placenta (9.3 mm) when compared to posterior (8.4 mm) and fundal location (4.2 mm) of the placenta.

Women with an amniotic fluid index less than 5 had a significantly higher error (− 12 mm, *p* value 0.056) when compared to normal liquor. Parity, maternal diabetes, and fetal presentation did not affect the error in ultrasonographic measurement of head circumference.

The difference between the sonographic and postnatal HC was also related to the mode of delivery, highest error in those who had instrumental delivery (− 13 mm). It was also noteworthy that when the HC was beyond 95th centile on ultrasound, the error detected postnatally was significant (− 14 mm vs. − 8 mm), though not statistically significant (*p* value 0.82).

## Discussion

In the present study, we evaluated the accuracy of measuring fetal head circumference on ultrasound by comparing it with the head circumference measured postnatally. We also looked into the factors affecting the accuracy of estimating sonographic HC.

Studies have shown that accurate measurement of fetal head circumference and fetal weight estimation by ultrasound are the essential prognostic parameters for many obstetric problems like evaluating the babies with fetal growth restrictions, fetal head anomalies in labor management by reducing the perinatal morbidity and mortality, and a beneficial tool for determining the further obstetric management [1, 9–11].

In the present study, the ultrasonographic head circumference was measured by eclipse that was traced all around the fetal skull within 5 days before delivery and was compared with actual HC postnatally measured 3 days after delivery. Overall, the mean sonographic HC was lower than the postnatal HC (Table 1). The sonographic underestimation of HC remained throughout gestation and became more striking as gestational age increased, reaching a mean difference of − 8.9 mm at gestational age beyond 37 weeks (Table 2). Ultrasonographic HC underestimated actual postnatal HC in 189 cases (87.5%). Ultrasonographic HC overestimated actual HC in 27 cases (12.5%). In a study by Melamed et al. [7], ultrasonographic fetal HC measurements taken within 3 days of delivery were compared with actual head circumference measurements taken postnatally within 2–6 h after delivery with a measuring tape. The current study showed significant underestimation of ultrasonographic HC measurements in comparison with the postnatally measured HC. Like in our study, Melamed et al. [7] also said that as the gestational age increased, the tendency for underestimation also increased.

We also studied the factors influencing the error in the estimation of ultrasonographic fetal HC. Gestational age, anterior location of the placenta, and low liquor of less than 5 had a statistically significant error in fetal HC, whereas parity, maternal diabetes, and fetal presentation did not affect the error in ultrasonographic measurement of head circumference.

A similar study conducted by Melamed et al. in 2011 [7] showed that the error in measuring fetal HC and the degree of underestimation were significantly higher in the case of gestational age ≥ 34 weeks, fundal placenta, high cephalic index and postnatal HC > 90th centile.

Wegrzyn et al. concluded that the influence of gestational age on measuring HC might be due to larger babies due to increased subcutaneous tissue [9].

Contrary to our results, Huber et al. in their systematic review on factors influencing the accuracy of fetal weight estimation (FWE) in preterm babies reported that maternal body mass index, gestational age, amniotic fluid index, presentation of the fetus, the presence of multiple fetuses and location of placenta do not seem to have an impact on the accuracy in fetal weight estimation. The influence of the sonologists expertise and fetal gender were discussed contentiously. The time interval between the fetal HC estimation and delivery, and varieties of formulas used to calculate FWE seem to have an evident effect on FWE accuracy [12].

In the present study, the difference between the sonographic and postnatal HC was also related to the mode of delivery with the highest error following instrumental vaginal delivery (− 13 mm). It was also noteworthy that when the HC was beyond 95th centile on ultrasound, the error detected postnatally was significant (− 14 mm vs. − 8 mm), though not statistically significant (*p *value 0.82). In their study, Melamed et al. [7] also stated that reduced ultrasound accuracy influenced delivery by vacuum extraction. Wegrzyn et al., in their study, stated that the error in HC depended on the mode of delivery. The error was least for cesarean sections and increased for vaginal deliveries and vacuum extraction [9].

Lipschuetz et al. [6], in their extensive retrospective analysis of 24,780 cases, studied if the fetal head, which interfaces with the birth canal, might impact obstetric outcomes more than birth weight. They analyzed the risk of unplanned cesarean or instrumental delivery, maternal and perinatal complications among those with HC or birth weight ≥ 95th centile. The study concluded that a large HC is strongly associated with unplanned cesarean and instrumental delivery than high birth weight. This result is significant to improve pre-labor counseling and clinical management of mothers with big babies. Hence, the accurate measurement of the fetal head circumference by ultrasound is more important than birth weight in assessing the mode of delivery.

Lipschuetz et al. [13], in another recent study published in 2018, analyzed the risk of undergoing unplanned cesarean in those with large fetal head circumference. He concluded that sonographic fetal HC > 35 cm measured within a week of delivery is an independent risk factor for an unplanned cesarean section but not for instrumental delivery. Fetal head circumference ≥ 35 cm and estimated fetal weight ≥ 3900 g significantly increased the risk of a prolonged second stage of labor.

Strength and limitations of the study: This is a prospective study. The ultrasound was performed by a single senior consultant. Hence, the interobserver errors in calculating fetal HC are minimized. However, since the sample size was very small, further research with a larger sample size is required to generalize our study results.

## Conclusion

The ultrasound estimation of fetal head circumference is associated with significant underestimation of the actual head circumference measured postnatally. The error in measuring fetal HC increases with advanced gestational age, low liquor, and anterior location of the placenta. Accurate measurement of fetal head circumference has important implications in specific clinical scenarios like monitoring pregnancy with fetal growth restriction, suspected fetal head growth abnormalities as well as labor management and perinatal outcomes. The error in fetal head circumference was more in those who had instrumental vaginal delivery. Hence, measuring accurate fetal head circumference in the last few days before delivery may be an important adjunct to estimated fetal weight in labor management and perinatal outcome.
